# The Peculiarities of Strain Relaxation in GaN/AlN Superlattices Grown on Vicinal GaN (0001) Substrate: Comparative XRD and AFM Study

**DOI:** 10.1186/s11671-016-1478-6

**Published:** 2016-05-17

**Authors:** Andrian V. Kuchuk, Serhii Kryvyi, Petro M. Lytvyn, Shibin Li, Vasyl P. Kladko, Morgan E. Ware, Yuriy I. Mazur, Nadiia V. Safryuk, Hryhorii V. Stanchu, Alexander E. Belyaev, Gregory J. Salamo

**Affiliations:** V. Lashkaryov Institute of Semiconductor Physics, National Academy of Sciences of Ukraine, Pr. Nauky 41, 03680 Kiev, Ukraine; Institute for Nanoscience and Engineering, University of Arkansas, West Dickson 731, Fayetteville, AR 72701 USA; State Key Laboratory of Electronic Thin Film and Integrated Devices, University of Electronic Science and Technology of China, 610054 Chengdu, China

**Keywords:** GaN/AlN, Superlattices, Strain relaxation, Crystallographic tilt, XRD, AFM

## Abstract

Superlattices (SLs) consisting of symmetric layers of GaN and AlN have been investigated. Detailed X-ray diffraction and reflectivity measurements demonstrate that the relaxation of built-up strain in the films generally increases with an increasing number of repetitions; however, an apparent relaxation for subcritical thickness SLs is explained through the accumulation of Nagai tilt at each interface of the SL. Additional atomic force microscopy measurements reveal surface pit densities which appear to correlate with the amount of residual strain in the films along with the appearance of cracks for SLs which have exceeded the critical thickness for plastic relaxation. These results indicate a total SL thickness beyond which growth may be limited for the formation of high-quality coherent crystal structures; however, they may indicate a growth window for the reduction of threading dislocations by controlled relaxation of the epilayers.

## Background

GaN/AlN superlattices (SLs) have been considered for high-performance photonic devices operating throughout the ultraviolet, visible, and infrared optical regions [1–3]. Among other factors such as growth conditions and design parameters, the structural and consequently optical properties of these SLs are strongly influenced by both the substrate type and the strain in the SLs. In general, this strain is a result of the large lattice mismatch between the GaN quantum well (QW) and the AlN barrier (2.5 % in-plane); however, an additional strain component results from the difference between the lattice spacing of the substrate and the averaged lattice spacing of the entire SL.

There has been significant research devoted to studying the influence of the substrate and buffer on the deformation and relaxation processes in GaN/Al(Ga)N SLs in recent years [4–14]. In particular, it has been demonstrated that both the Al mole fraction and the buffer layer type (tensile-strained GaN or compressive-strained AlGaN) have strong influences on the misfit relaxation process in 40-period, 7/4-nm, GaN/Al_*x*_Ga_1 − *x*_N SLs [6, 7]. However, a minimization of strain relaxation by growth of both GaN and AlN under Ga excess conditions was shown for GaN/AlN (1.5/3 nm) SLs grown on both AlN- and GaN-on-sapphire templates [5]. A bimodal strain relaxation of GaN/AlN short-period SL structures independent of the type of template (GaN-thick- or AlN-thin-on-sapphire) was observed in [8, 9]. This is contrasted by the data presented in [10], which unambiguously demonstrates that the structural quality of a 10-period GaN/AlGaN SL is limited by the structural properties of the GaN substrate. This can be improved upon as seen in [12, 13] by growing on non-polar free-standing GaN substrates. In particular, for growth of non-polar *m*-plane GaN/AlGaN multi-QWs, extended defects introduced by the epitaxial process, such as stacking faults or dislocations, were not observed [13].

A commonly used technique to improve the crystal quality of III-nitride heteroepitaxial layers is to grow on miscut substrates [15–20], resulting, however, also in a crystallographic tilt of the epilayers. This has been observed for GaN films grown on both vicinal Al_2_O_3_ and 6H-SiC substrates [15, 16]. Here, the relationship between the tilt of the GaN lattice and the offcut angles and the surface steps of the substrate was directly established. The influence of the *c*-plane vicinal GaN substrates on the crystallographic orientation and deformation of InGaN layers was shown in [17]. The crystallographic tilting of GaN/AlN layers grown on Si (111) substrates with different miscut angles towards the [110] direction was reported in [18, 19]. A common result to these studies is a tilting of the lattice planes of the epitaxial layer with respect to the lattice planes of the substrate in addition to what would be expected by simple geometric arguments of the miscut and step density. This is the so-called *Nagai tilt* [20]. Apart from this, the impact of the substrate miscut (via influence on misfit dislocation) on the epilayer quality has been demonstrated. As for the GaN/AlN SLs, it was found that growth on vicinal Al_2_O_3_ (0001) substrates shows uniform layer structures with abrupt interfaces and good periodicity [21]. It was demonstrated that the use of an appropriate vicinal substrate with an angle of ~0.5° improves the quality of GaN/AlN SLs, leaving an extremely flat surface without any growth-induced defects.

In this work, we present the peculiarities of crystallographic tilting and strain relaxation in GaN/AlN SLs grown on vicinal GaN (0001) surfaces by plasma-assisted molecular beam epitaxy (PAMBE). Structural properties and the evolution of the deformation state as a result of changes in the number of periods in GaN/AlN SLs are investigated by high-resolution X-ray diffraction (HRXRD), X-ray reflectivity (XRR), and atomic force microscopy (AFM) techniques.

## Methods

The GaN/AlN SLs were grown by PAMBE under an activated nitrogen plasma flux in a metal-rich regime at a substrate temperature of ~760 °C. Three SLs consisting of GaN/AlN (5/5 nm) periods capped with an additional GaN (10 nm) layer were grown on GaN buffer layers (100 nm) deposited on GaN (4 μm)/*c*-Al_2_O_3_ templates. The numbers of periods were 5 (sample number S5), 10 (S10), and 20 (S20). The evolution of the deformation state and structural properties of the SLs were examined ex situ using PANalytical X’Pert Pro MRD XL (X’Pert, PANalytical B.V., Almelo, The Netherlands) and NanoScope IIIa Dimension 3000™ (Digital Instruments, Inc., Tonawanda, NY, USA) systems for HRXRD and AFM characterization. For HRXRD, we used a standard four-bounce Ge (220) monochromator and a three-bounce (022) channel-cut Ge analyzer crystal along with a 1.6-kW X-ray tube with CuKα_1_ radiation and vertical line focus.

## Results and Discussion

### XRD Characterization

**Fig. 1:**
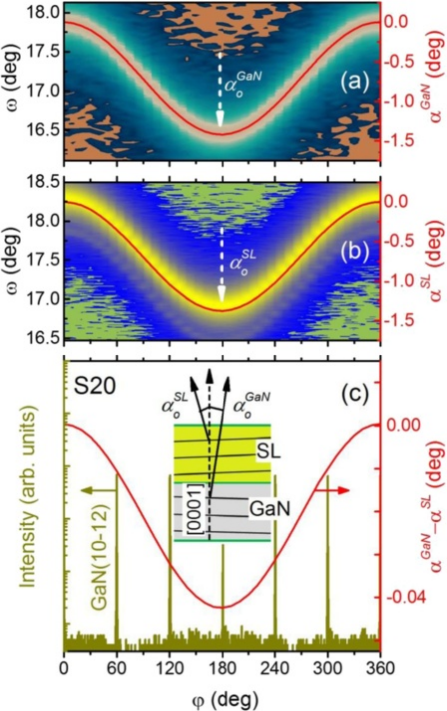
The experimental *ω* − *φ* 2D intensity scattering maps of *a* GaN (0002) and *b* SL (0002) reflections for S20. The *red curves* are the fitted offset angles of *α* ^GaN^ and *α* ^SL^ with Eq. (1). The subtracted *α* ^GaN^ − *α* ^SL^ curve along with the *φ*-scan for GaN $\left(10\overline{1}2\right)$ reflection is shown in *c*. The *inset* demonstrates the misorientation angles relative to the surface normal direction

To accurately determine the misorientation angles of the GaN substrates $\left({\alpha}_0^{\mathrm{GaN}}\right)$ and the GaN/AlN SLs $\left({\alpha}_0^{\mathrm{SL}}\right)$, i.e., the crystallographic tilts of the GaN and SL [0001] axes from the surface normal direction, *ω* − *φ* 2D intensity scattering maps for the GaN (0002) and the SL (0002) reflections were measured in the azimuthal scanning range of *φ* = 0° to 360°, with a step size of 10°. Typical *ω* − *φ* 2D maps for S20 are shown in Fig. 1(a, b). From the position, *ω*, of the diffraction maximum as a function of the azimuthal angle *φ*, i.e., *ω*(*φ*), we can determine the offset angle as a function of azimuth, *α*(*φ*) = *ω*(*φ*) − *θ*_B_ (where *θ*_B_ is the Bragg angle).

The misorientation angle, *α*_0_, of a target lattice plane was found by fitting the experimental function, *α*(*φ*), with the following equation:

1 $$ \tan \left(\alpha \left(\varphi \right)+{c}_1\right)= \cos \left(\varphi +{c}_2\right)\times \tan \left({\alpha}_0\right) $$

where *c*_1_ and *c*_2_ are the fitting parameters [19]. The fitted ${\alpha}_0^{\mathrm{GaN}}$ and ${\alpha}_0^{\mathrm{SL}}$ values along with the extracted crystallographic tilt ($\varDelta {\alpha}_0^{\mathrm{SL}}={\alpha}_0^{\mathrm{GaN}}-{\alpha}_0^{\mathrm{SL}}$) of the SL are summarized in Table 1 for all samples. As can be seen, the misorientation of the GaN (0002) is 0.69° ± 0.015° and that of the SL (0002) is ~0.67° ± 0.015°, both tilted to the same azimuth without any significant phase shift (i.e., difference in *φ*). To determine the crystallographic direction of the misorientation, the *φ*-scan of GaN $\left(10\overline{1}2\right)$ reflection was measured in the same azimuthal scanning range as above. As can be seen from Fig. 1(c), the orientation of the crystallographic tilts of GaN and the SL follows the same crystallographic direction, along the GaN $\left[10\overline{1}0\right]$. It should be noted that an additional tilt $\left(\varDelta {\alpha}_0^{\mathrm{SL}}\right)$ of the lattice *c*-planes of the SLs with respect to the lattice *c*-planes of the GaN substrates is not the same for all samples (see Table 1). In order to establish the relationship between the tilt of the lattice of SLs and the offcut angles of GaN substrate, their lattice parameters need to be taken into account.

**Table 1:** The misorientation angles of GaN substrate and SLs for each sample. The fitted ${\alpha}_0^{\mathrm{GaN}}$ and ${\alpha}_0^{\mathrm{SL}}$ values are given along with the experimental ($\varDelta {\alpha}_0^{\mathrm{SL}}$) and calculated ($\varDelta {\alpha}_0^{\mathrm{SL}}\kern0.5em \left(\mathrm{Nagai}\right)$) crystallographic tilt of SLs

Sample	${\alpha}_0^{\mathrm{GaN}}$ (°)	${\alpha}_0^{\mathrm{SL}}$ (°)	$\varDelta {\alpha}_0^{\mathrm{SL}}$ (°)	$\varDelta {\alpha}_0^{\mathrm{SL}}\kern0.5em \left(\mathrm{Nagai}\right)$ (°)
S5	0.676	0.658	−0.018	−0.014
S10	0.684	0.667	−0.017	−0.014
S20	0.710	0.688	−0.022	−0.015

**Fig. 2:**
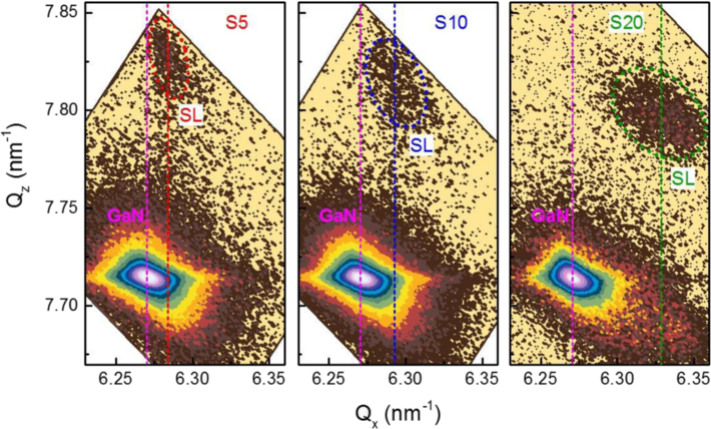
The $\left(11\overline{2}4\right)$ RSMs of GaN buffer layers and GaN/AlN SLs for S5, S10, and S20. The *vertical dashed lines* indicate the *Q*_*x*_ positions for GaN and SLs

In order to study the evolution of the deformation state and structural parameters of GaN/AlN SLs, reciprocal space mapping (RSM) was used firstly. To avoid errors by characterization of tilted layers, we used an approach well described in [17]: we mount the sample in such a way that the direction of the miscut is perpendicular to the diffraction plane. The interplanar distances of asymmetric planes measured in such arrangement of the sample are not influenced by the tilt. All samples were measured in the vicinity of the GaN $\left(11\overline{2}4\right)$ reflection, and the results are shown in Fig. 2. It is seen that for all samples, the SL peaks are not vertically aligned with the GaN peak. This arrangement of the GaN and SL peaks on the *Q*_*x*_-axis indicates that the SL structures are not fully strained to the GaN buffer layer (*a*_SLs_ ≠ *a*_GaN_). Moreover, the arrangement of the SL peaks on the *Q*_*z*_-axis indicates an evolution of the out-of-plane lattice parameter by changing the number of periods in the SLs. Therefore, the mean strain of the SLs must depend on the number of SL periods. The measured values of the in-plane lattice parameters for SL and GaN buffers extracted from the asymmetrical RSMs are listed in Table 2. Here, by comparing *a*_SLs_ and *a*_GaN_, we can conclude that the in-plane strain relaxation of a SL increases by increasing the number of SL periods. This leads to a change in the relaxation degree of individual layers of the SLs, i.e., the GaN QW and AlN barrier layers. To define the relaxation values of the GaN QW and AlN barrier layers, we used Eq. (2) [22].

**Table 2:** Structural parameters for SL layers and GaN substrate obtained from HRXRD data for the different samples investigated

Sample	RSM $\left(11\overline{2}4\right)$			*ω*/2*θ* (0002)		*ω* (0002)	
	*a* (nm)	*R*_GaN_ (%)	*R*_AlN_ (%)	*T*_SL_ (nm)	*t*_GaN_/*t*_AlN_ (nm)	Δ*ω* (arcsec)	*N*_screw_ (×10^8^ cm^2^)
S5	0.3183 ± 0.0001	92	8	9.9 ± 0.15	4.9/5	477.6	4.58
S10	0.3177 ± 0.0001	83	17	9.7 ± 0.20	4.8/4.9	843.2	14.3
S20	0.3158 ± 0.0001	59	41	9.45 ± 0.05	4.35/5.1	406.4	3.32
GaN_templ._	0.31878 ± 0.00002	99.95				238.9	1.12

2 $$ {R}_{\mathrm{well},\ \mathrm{barrier}}=100\times \raisebox{1ex}{$\left({a}_{\mathrm{SL}} - {a}_{\mathrm{AlN},\ \mathrm{G}\mathrm{a}\mathrm{N}}\right)$}\!\left/ \!\raisebox{-1ex}{$\left({a}_{\mathrm{GaN},\ \mathrm{A}\mathrm{l}\mathrm{N}} - {a}_{\mathrm{AlN},\ \mathrm{G}\mathrm{a}\mathrm{N}}\right)$}\right. $$

where *a*_SL_ = *a*_well_ = *a*_barrier_ and *a*_GaN_ and *a*_AlN_ are the bulk relaxed lattice parameters of GaN and AlN, respectively. If we assume that the GaN QW and the AlN barrier layers in the SLs are mutually lattice-matched with each other, then the sum of the relaxation values *R*_well_ + *R*_barrier_ = 100 %. Thus, a change in the relaxation degree of the entire SL by increasing the number of SL periods leads to the decrease and increase of relaxation degree of the GaN QW and AlN barrier, respectively (see Table 2).

**Fig. 3:**
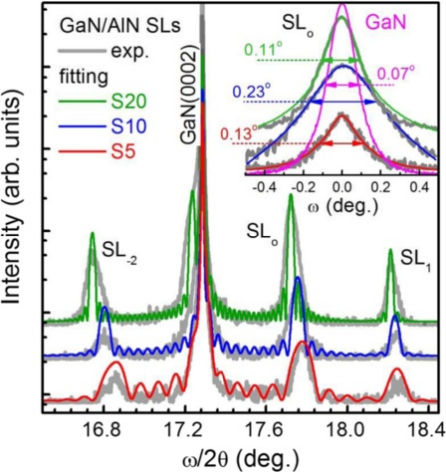
The experimental (*gray curves*) and fitted (*color curves*) (0002) *ω*/2*θ* XRD spectra for each sample. The *inset* represents the (0002) *ω*-scans for the zero-order satellite peak of SLs along with the FWHM (Δ*ω*) presented for each sample

Taking into account the relaxation values, *R*_well, barrier_, we simulated the HRXRD (0002) *ω*/2*θ*-scan using the X’Pert Epitaxy software package (see Fig. 3). Firstly, we determine directly the SL period thickness from the separation angle of the SL satellite peaks. Next, by varying the GaN QW and AlN barrier thicknesses at fixed relaxation values, we achieved a good fitting of the experimental HRXRD spectra. The extracted SL periods (*T*_SL_) and the GaN QW and AlN barrier thicknesses (*t*_GaN_/*t*_AlN_) are given in Table 2. The simulations fit the experimental spectra quite well, and we observed some differences between these measured and design thicknesses of the SL layers.

**Fig. 4:**
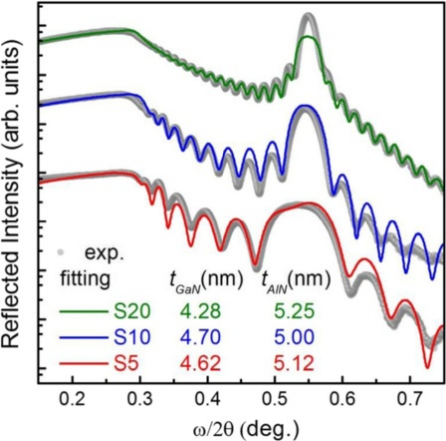
The XRR profiles of S20 (*green*), S10 (*blue*), and S5 (*red*). The *gray curves* are the experimental XRR profiles for each sample

In order to confirm the *T*_SL_, *t*_GaN_, and *t*_AlN_ values obtained from the simulation of the (0002) *ω*/2*θ* XRD spectra, we additionally measured the *ω*/2*θ* XRR profiles for each sample. This method is not sensitive to the deformation of the lattice parameter. The thickness oscillations in XRR, i.e., Kiessig fringes, are caused by the interference of the waves reflected at the layer surfaces; therefore, the oscillation period determines the thicknesses associated with the well, barrier, and SL period, respectively. To assess these values, the XRR experimental scans were fitted with the X’Pert Reflectivity software package (see Fig. 4). Comparing these two techniques, it was observed that there was a relatively small disparity in the thickness of the calculated layers between the two methods, which may be easily accounted for by experimental error. However, the trends in thickness change remain the same. The observed thickness reduction mainly for the GaN QW can be explained by the following [23, 24]: (i) the Al-N binding energy is much higher than the Ga-N binding energy, (ii) the exchange between the Ga atoms of the QW and the Al adatoms of the barrier is thermally activated, and (iii) the strain in the GaN QWs influences the Al-Ga exchange mechanism.

In order to more deeply study the evolution of the structural parameters due to the changing number of periods in the SLs, we measured the *ω*-scan for the zero-order satellite peaks (see the inset in Fig. 3). As we can see, the full width at half maximum (Δ*ω*) of the (0002) rocking curves for the SL peaks is larger than that for the GaN buffer peak for all samples. By using the equation *N*_s_ = *Δω*^2^_(0002)_/(4.35 × |**b**_s_|^2^), where **b**_s_ is Burger’s vector of screw-type threading dislocations (TDs), we calculate the density of screw-type TDs (*N*_screw_). The pure screw-type TD has a Burger’s vector, |**b**_**s**_| = *c* = 0.51851 nm, in the [0001] direction. As can be seen from Table 2, *N*_screw_ for the SLs is higher than that for the GaN buffer for all samples. Moreover, the non-monotonic change in the density of TDs with the changing number of periods in SLs is evident. This indicates that some critical thickness has been exceeded in the growth of S20.

The densities of TDs in the SLs appear to correlate directly with the strain in the SLs. In addition to the lattice mismatch between the GaN/AlN layers of the SL and the GaN substrate, determined through the ratio of layer thicknesses in the SL (*t*_GaN_/*t*_AlN_), the strain also accumulates in the film through an increasing number of periods. The critical thickness for plastic strain relaxation depends, finally, on the accumulated elastic energy, the surface free energy, and the energy required for the generation of a dislocation. In the case of the GaN/AlN SL deposited on GaN with *t*_AlN_/*t*_GaN_ = 2 [5], it was shown experimentally that the average in-plane lattice parameter decreases gradually as strain builds up and dislocations are generated and reach a stable value after about 20 SL periods with a critical thickness of ~90 nm. For our SLs with *t*_AlN_/*t*_GaN_ ∼ 1, the non-monotonic change in the density of TDs with the evolution of the deformation state in SLs indicates that the critical thickness for plastic relaxation is exceeded for films greater than 10 periods or a total thickness of ~97 nm. Below this thickness, like for S5 (~50 nm), the elastic strain is due entirely to lattice mismatch. Above the critical thickness like S20 (~190 nm), plastic relaxation through misfit dislocation generation must be considered in the evaluation of the total strain in addition to the lattice mismatch. Even after these considerations, the resulting strain cannot be explained. We must also take into account the strain relief due to the non-ideality of the crystal orientation, i.e., the miscut.

Finally, in order to include the miscut in the analysis, we must consider the out-of-plane lattice parameters of the SLs (*c*_SL_) and GaN (*c*_GaN_) which allows us to calculate the Nagai tilt angle, $\varDelta {\alpha}_0^{\mathrm{SL}}\ \left(\mathrm{Nagai}\right)$, using the following equation [20]:

3 $$ \tan \varDelta {\alpha}_0^{\mathrm{SL}}\ \left(\mathrm{Nagai}\right)/ \tan {\alpha}_0^{\mathrm{GaN}}=\left({c}_{\mathrm{SL}}-{c}_{\mathrm{GaN}}\right)/{c}_{\mathrm{GaN}} $$

As can be seen from Table 1, for all SL samples, the measured tilt angles, $\varDelta {\alpha}_0^{\mathrm{SL}}$, are larger than the Nagai angle predicted by Eq. (3). Huang et al. [15] reported that the tilt angle of the GaN layer obeys the Nagai model if the misorientation angles of the sapphire substrate are small. The small angle miscut is in fact the required approximation for the classical Nagai theory. However, despite the small misorientation angles of our GaN substrate, the classic Nagai theory does not appear to be valid for our samples. First of all, the small miscut approximation of the classical Nagai model only considers the out-of-plane lattice mismatch between two ideal crystal lattices at one interface, which would treat a superlattice as some average alloy. In general, the multiple layers of the SLs require a more complicated consideration of the difference in the out-of-plane lattice mismatch as well as a consideration of the in-plane mismatch. This was explicitly demonstrated for GaN/AlN layers grown on Si (111) substrates with different misorientation angles [18, 19]. Additionally, these models only consider tilting or lattice misorientation of single epitaxial interfaces. For our GaN/AlN SLs grown on vicinal GaN (0001) substrate, the deposition of each additional layer is characterized by (i) the presence of elastic and plastic relaxation components [5], due to the in-plane lattice mismatch with the resulting layers below it, and (ii) the tilting or a triclinic unit cell deformation, due to the out-of-plane lattice mismatch with the resulting layers below. The triclinic deformation of the unit cells of fully strained InGaN grown on vicinal GaN (0001) substrates was reported in [17]. A full analysis including these considerations is beyond the scope of this paper but should explain the result that the SL peak of S5 is not vertically aligned with the GaN substrate peak (Fig. 2(a)), even though S5 is below the critical thickness for plastic relaxation and is pseudomorphic with and fully strained to the substrate. The final result of this Nagai tilt is only a small additional elastic relaxation component at an interface, but here, we have shown that this elastic relaxation pathway can be considerable after the addition of many interfaces through the growth of a SL.

### AFM Characterization

**Fig. 5:**
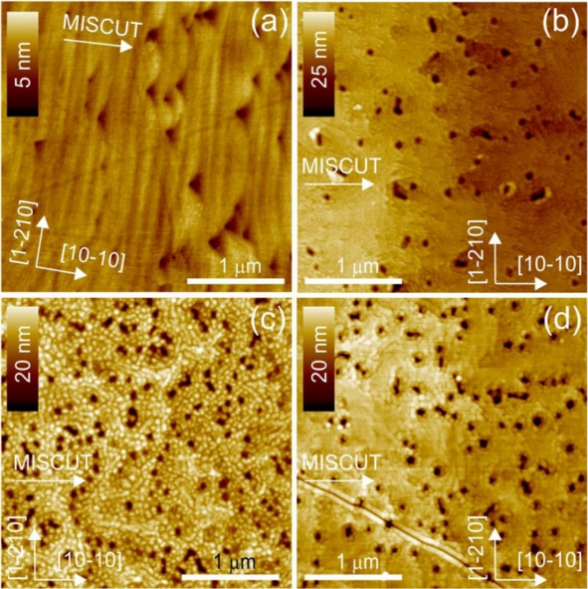
AFM image of the surfaces of the substrate GaN/Al_2_O_3_ (**a**) and the SL structures of AlN/GaN with 5, 10, and 20 periods, respectively, in **b**–**d**. The *arrows* indicate crystallographic directions and the direction of misorientation of substrate

**Fig. 6:**
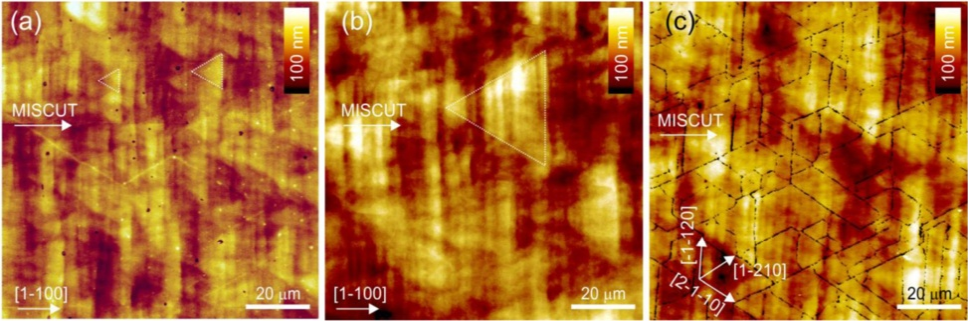
AFM images of surfaces of the AlN/GaN SL structures with 5, 10, and 20 periods, respectively, in **a**–**c**. The *dotted lines* mark the triangular-shaped areas of anisotropic step-flow growth resulting from the long-range pinning effects of the surface-terminated dislocations

The topographic features of the epitaxially grown surface indirectly carry information about mechanisms of growth and relaxation, residual deformations, and structural defects. Therefore, we have used atomic force microscopy to analyze the surface features of the samples. To ensure high sensitivity and resolution, measurements were carried out in the tapping mode using silicon tips with a nominal tip radius of less than 10 nm. The typical topography of GaN-on-sapphire template substrate and cap layer of the multilayer superlattice AlN/GaN structures is shown in Fig. 5. Here, we see that the surface is densely covered with depressions, which are evidently the emergence of mixed or pure screw TDs with a Burger’s vector, |***b***_m_|^2^ = (1/3 × *a*)^2^ + *c*^2^, in the $\left[11\overline{2}3\right]$ direction, or |***b***_s_| = *c*, in the [0001] direction [25]. The formation of pits at TD cores is possible due to the strain energy density associated with surface-terminated threading dislocations being equivalent to a line of tension directed into the material [26]. All surfaces show characteristic features which result from the 2D step-flow growth mechanism of Ga-rich growth, but each sample has individual features. In the GaN buffer (Fig. 5a), narrow terraces are observed with a width of ~129 nm and a height of ~0.53 nm, which is very nearly the same as the bilayer step height of GaN (0.518 nm). The direction of terraces, of course, coincides with the direction of the misorientation of the sapphire substrate, while the surfaces of the terraces are atomically smooth. Dislocations act to pin the step flow of the terraces forming the characteristic triangular shapes seen at the step edges and resulting in an observed density of dislocations of ~0.5 · 10^9^ cm^−2^. Deposition of the SL structures significantly increased the width of the terraces. These range from ~3500 nm in the 5-period SL sample to ~2500 nm in the 20-period SL sample and are evidence of significant step bunching (Fig. 6). This step bunching also results in terrace heights which range from ~8 nm in the 5-period SL to ~5 nm in the 20-period SL. When averaging the width/height of the terraces over 100 × 100 μm areas, the misorientation angle of the surface layers is 0.08°, 0.06°, and 0.10° for S5, S10, and S20, respectively. For the GaN buffer layer, the misorientation is much larger than ~0.23°.

In addition, the surfaces of the terraces cease to be atomically smooth and the nanograin substructures appear (Fig. 5b–d). Nanograins of about 20 nm are located in the vicinity of the dislocation pits on the 5-period SL. For the 20-period SL sample, the nanograin size is ~40 nm. However, the nanogranularity of the terraces is much more significant and in fact more uniform in the 10-period SL sample where the typical grain size is 50–60 nm and the grains evenly cover the entire surface. Looking, however, at the surfaces over a larger scale (Fig. 6a, b), we find the formation of triangular-shaped areas which are caused again by the blocking of the isotropic step-flow growth similar to the GaN buffer layer [25, 27, 28].

According to AFM measurements of the SL samples, S5 and S10 display an increased density of TDs of 1.2 × 10^9^ and 1.7 × 10^9^ cm^−2^, respectively. However, S20 exhibits a dislocation density of only 0.7 × 10^9^ cm^−2^, which is commensurate with the density of dislocations in the buffer layer of GaN. At the same time, though, this reduction in the dislocation density is accompanied by an observed cracking of structure (Fig. 6c). It is known [29, 30] that this type of crack morphology is attributed to the combination of cleavage of $\left(10\overline{1}0\right)$-like planes in GaN and parting of $\left(11\overline{2}0\right)$-like planes in α-Al_2_O_3_, because cracking in the substrate and the epitaxial layer will occur simultaneously. With cracks generated at the (0001) GaN/(0001) α-Al_2_O_3_ interface, they run dominantly along the GaN $\left[11\overline{2}0\right]$ and α-Al_2_O_3_$\left[10\overline{1}0\right]$ directions. It should be noted that the tendency of changes in density of TDs from AFM correlates well with changes in density of screw-type TDs from HRXRD data. The comparison of the absolute values is not possible, because the AFM gives the density of both screw- and edge-type TDs, but from HRXRD, we extract only the density of screw-type TDs. Moreover, since the cracking of the structure can influence the full width at half maximum of the XRD rocking curves, the density of dislocations extracted from HRXRD can be overestimated.

The experimental data obtained illustrate the progress of a number of structural relaxation processes, each of which is manifested to a different degree depending on the level of strain in the SLs. The most significant of them is cracking and generation of dislocations.

## Conclusions

In this work, we have investigated strain relaxation through the growth and analysis of GaN/AlN SLs. SLs were grown nominally with symmetric 5-nm GaN wells and 5-nm AlN barriers repeated 5, 10, and 20 times. Detailed X-ray diffraction and reflectivity measurements demonstrated that the relaxation of the films generally increased with an increasing number of repetitions; however, the additional consideration of a Nagai tilt at each interface can explain the small apparent relaxation for the 5-period SL which is considered to be completely strained to the substrate. Additionally hidden is a transition to a different relaxation mechanism as the growth exceeds a critical thickness for relaxation, as AFM measurements revealed a sharp drop in the density of pits and associated threading dislocations for the 20-period sample. At the same time, an increase in the number of observed cracks was found for the 20-period sample. These results indicate a total SL thickness beyond which growth may be limited for the formation of high-quality coherent crystal structures; however, they may indicate a growth window for the reduction of threading dislocations by controlled relaxation of the epilayers.
